# Trends of land use land cover dynamics of *Sheka* biosphere reserve, A case of *Shato* core area, Southwest Ethiopia

**DOI:** 10.1371/journal.pone.0287830

**Published:** 2023-11-16

**Authors:** Workaferahu Ameneshewa, Yechale Kebede, Dikaso Unbushe, Abiyot Legesse

**Affiliations:** 1 Department of Surveying Engineering, College of Engineering and Technology, Mizan-Tepi University, Tepi, Ethiopia; 2 Department of Geography and Environmental Studies, College of Social Science, Arbaminch University, Arba Minch, Ethiopia; 3 Department of Biology, College of Natural Science, Wolaita Sodo University, Sodo, Ethiopia; 4 Department of Geography and Environmental Studies, College of Social Science, Dilla University, Dilla, Ethiopia; Van Lang University: Truong Dai hoc Van Lang, VIET NAM

## Abstract

The usage of land use and land cover change information has significantly risen as a result of the requirement for relevant data for environmental monitoring, modeling, and planning. The main objective of the study is to analyze the trend of land use and land cover dynamics in *Sheka* biosphere reserve: A Case of *Shato* Core Area, Southwest Ethiopia. To map the land use and land cover, supervised classifications were used, and an accuracy evaluation was conducted. Information on the trend of land use and land cover change was obtained using the IDRISI software’s land change modeler. Results showed that about 308.29ha (56.7%) of wetland and 3,215.6ha (19.6%) of natural forest were converted to other land use types in the last 30 years. Plantation and rural settlement increased by 2,234.3ha (10.2%) and 1289.6ha (6.6%) respectively from 1991–2021. *Sheka* biosphere reserve was registered with UNESCO in 2012 and *Shato* is one of the core areas. It covers 5023.3ha (25.5%) of the study area. However, 1482ha (7.5%) were given to coffee plantations, and currently, only 3,541ha (18%) are left. The main drivers of land cover changes were attributed to large-scale agricultural intensification and its induced expansions of rural settlements in and around the *Shato* core area. The overall accuracy and kappa statistics for 1991, 2006, and 2021 were 74% (0.74), 81% (0.81), and 81.2% (0.812), respectively. Thus, land use and land cover change trend mapping and analysis play a crucial role in conservation planning and habitat monitoring. The study concluded that wetland and forestland conversions in the study area are decreasing overwhelmingly and need serious intervention mechanisms to tackle the loss of biodiversity in the *Shato* core area.

## 1. Introduction

### 1.1 Background

The amount of land covered by forests is a key indicator of the state of the ecosystem, and humans are now in charge of modifying this environment. The global rate of net annual forest loss was shown by FRA (2015) to have decreased from 7.3 to 3.3 million hectares annually, a reduction of more than 50% over the time periods 1990 to 2000 and 2010 to 2015 as a result of a combination of forest area loss and gain in some countries [1].

In Africa, forests cover about 21.4% of the land area which corresponds to 674 million hectares, whereas Eastern Africa alone covers approximately (13%) of the land area under the forests and woodlands based on the FAO report [2]. However, the net forest loss in Africa has increased in three decades since 1990, with the highest annual net forest loss occurring between 2010 and 2020, at 3.9 million ha [3]. Besides, FAO’s (2010) report depicted that the forest cover of Ethiopia shows a decline from 15.11 million ha in 1990 to 12.2 million ha in 2010, during which 2.65% of the forest cover was deforested [2]. In addition, planted forest cover of Ethiopia is estimated to be 15.7%, which shows increments because of a national wide reforestation program launched in 2000.

Forest has important environmental benefits, such as benefits of regional climate, biodiversity, greenhouse effects, purifying the air and water pollution [4]. However, management failures have been caused by a lack of knowledge about the entire values obtained from the forest as well as underestimating the benefits.

Ethiopia’s rich forest biodiversity is under serious threat from deforestation, land degradation, over-exploitation, overgrazing, habitat loss, and invasive species [5]. In most cases, the major destructive factor of plant diversity is deforestation caused by small and large-scale agricultural expansion, livestock grazing, and fuel wood scavenging [6, 7]. The other threats to the plant biodiversity of the country are unsustainable utilization of natural resources, land degradation, habitat loss and fragmentation, wetland destruction, and climate change [8]. The indirect threats comprise gaps in the application of forest policy and regulations; tenure/unclear forest user rights; lack of private investment in forestry development; population growth; inadequate land use planning and participatory forest management (PFM) related implementation gaps [9].

Most of the vast remaining forest resources in Ethiopia are found in the southwest, especially in the *Wellega*, *Illubabo*r, *Jimma*, *Shek*a, *Keff*a, and *Bench-Sheko* zones. According to estimates, closed alpine forest cover in southwestern Ethiopia declined from 40% between 1971 and 1975 to 18% in 1997 [10].

The *Sheka* Forest Biosphere Reserve in southwestern Ethiopia is located in a hotspot of mountain biodiversity in East Africa. It is one of the largest continuous forests in the country [1]). In recent decades, these valuable assets have been under tremendous pressure due to the emergence of shifts to plantations, settlements, and agriculture [10]. They added that traditional community tenure rights, their management methods, and the religious significance of cultural forests to the populace were not recognized by government policies and legislation. This is often due to basically change of forests to agribusiness especially monoculture plantations of coffee and tea. Between 1987 and 2005, high deforestation rates of 36% for tea plantations were noted in four areas of the *Sheka* zone, while earlier in 1987, there were no tea ventures in the zone [11].

Due to its establishment in a valuable forest area, the East African Tea Plantation was the most devastating to the region’s biodiversity [12]. Besides, *Haile* and *Alem* coffee plantation Plc. had taken over 1400 hector of land in the center of *Shato* core area “Fig 7”.

Even though large-scale agriculture in particular has had a "catalytic" role in attaining rapid economic growth, the sector often has adverse impacts on society and their environment [13]. The conversion of primary forests to agriculture is considered an irreversible loss, particularly regarding its biodiversity functions. And, Changes to plantations are also driven by a loss of conventional bee-farming practices and the loss of livelihood of the nearby community [14, 15].

With no prior environmental impact assessment (EIA), many plantations in forests have been established. Authorities, such as the Environmental Protection Agency, currently accept project EIAs prepared by investors on the basis of trust and without verification [16].

The locals’ perception and respect of taboos, cultural forests, and sacred sites have changed as a result of land clearing for commercial farming. Furthermore, the area’s forests have degraded due to investor expansion of coffee and tea on farmers’ land via an out-grower technique. Investors have trained farmers and given them tens of thousands of tea seedlings to encourage this expansion [16, 17].

The study area is a hotspot for biodiversity as well as the source of the *Didessa* River and the two largest tributaries of the Blue Nile, the *Omo* and *Baro*, which come in second and third place in terms of flow, respectively. Moreover, the valleys of these rivers are where 50% of the country’s irrigable land is found [14]. In addition to national benefits, the *Baro* and *Didessa* Rivers have been contributing to irrigation development in the Sudan [18]. These forests’ worldwide significance as well as their local, national, and cross-border advantages must be thoroughly recorded [15]. Furthermore, the study area has not received any attention, and no studies of the vegetation or the mapping of the forest cover in the *Shato* core area have been conducted.

The majority of earlier studies, however, emphasized on the economic benefits of forests, the effects of forest loss on the neighborhood community, the development of a dynamic framework to compare the best combination of forests and tea plants for land use [14] and ethnobotanical inventory [19]. The remaining focused entirely on the composition, structure, and regeneration status of the floral community [20] and gaining insights into the drivers of land use change in relation to land degradation environmental history of the forested parts of southwest Ethiopia [21].

Land use and land cover changes in the study area have not been the subject of any noteworthy research. The only prior study by [11] largely focused on investment-induced land use expansion toward the dense forest, but because it was conducted earlier than 14 years ago and did not take household-induced forest cover change into account, it didn’t reflect current land use/land cover dynamics in the study area. Consequently, this study has taken into account both household and investment-induced changes in the forest cover in the study area and aimed to fulfill the following objectives; detect the trend of LULC dynamics in the study area; quantify area changes under each land use/land cover categories; ascertain the threat on forest resource that currently exists in the study area.

## 2. Material and methods

### 2.1. Study area descriptions

**Fig 1:**
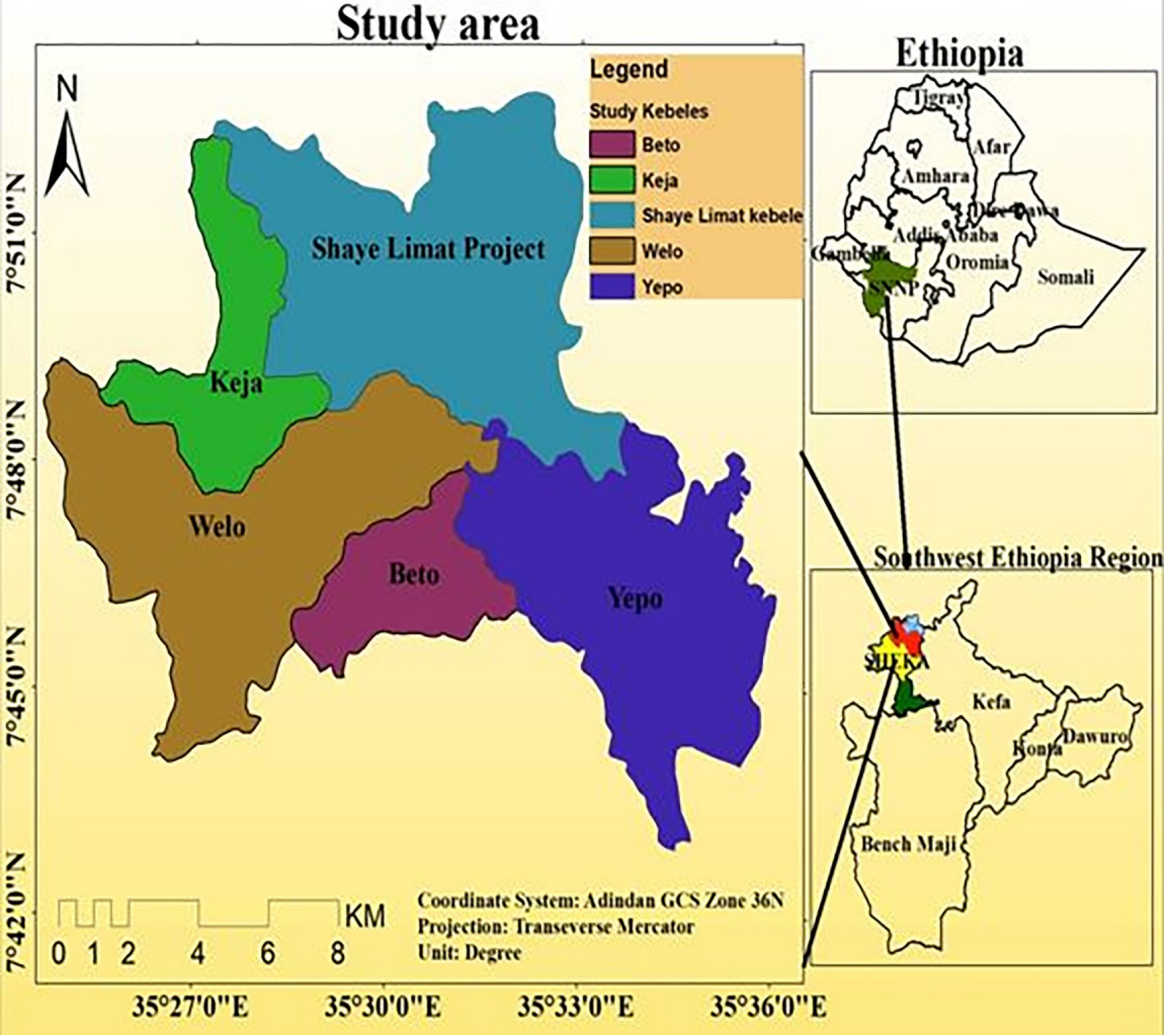
Map of the study area. Source; Central Statistical Authority.

The study was conducted in *Masha* woreda of *Sheka* zone situated within the South-west Ethiopia regional state, bordering both the *Gambela* region in the West and the Oromia region in the North and East. It consists of three *Woredas* (*Yeki*, *Anderacha*, and *Masha*). Woreda’s terrain is rugged and made up of mountainous areas that have influenced agricultural practices and settlement patterns. The woreda forest covers a large area and has high biodiversity. Specifically, the topography of the study area includes flat areas, rough terrain, plateaus, and areas with steep and gentle slopes. This study emphasizes five kebeles of *masha woreda*. The five *kebeles* of Masha Woreda were discussed in this study. Astronomically, those *kebele* locate Geographic coordinate system of Adindan 7°42’0"-7°51’0"N and 35°27’0"-35°36’0"E “Fig 1”. Attitudinally, those *kebele*s lie between 1600-2400m and with a total area of 19, 961ha.

### 2.2. Background

The *Sheka* zone is one of the few zones in Ethiopia, where traditional beliefs and ecological knowledge have aided the conservation of forests up to now. As a result, forest areas in the *Sheka* zone are part of the largest un-fragmented forest remaining in the country, and by far the largest in the Horn of Africa [10]. Especially, in *masha woreda* and *Andracha woreda* local communities have developed a long tradition of sustainable forest management, called ***Kobo*** forestry, mainly for honey production though also for harvesting wild coffee and cardamom.

*Masha Woreda* has 19 *Kebeles* and one chartered town called *Masha* and it is the capital of Sheka Zone. The *woreda* is bounded to the West by Sele- Nonno Woreda of Oromia region, to the South by *Diddo-Lallo Woreda* of Oromia region, and to the North by Andracha *Woreda* of *Sheka* Zone and has a total land area of 90,802.82 hectares. Out of this land area, about 23.9% is cultivated, 2.8% is grazing land, 40.5% is covered by forest, 5.5% is arable land, 5.9% is non-arable land and 21.4% is settled land area [22].

Eleven major soil types cover about 87% of the land. Cambisols, which cover 13% of the country, are the most common soil type, followed by lithosols covering 12%. Other soil types include vertisols (10%), xerosols (8.5%), acrisols (8%), luvisols (6%), xelonchakes (5%), regosols (4%) and yermosols (3%) [23]. The major reference soil groups of the south-western highland plateaus are Nitisols, Vertisols, Leptosols, Regosols, Cambisols, and Acrisols. Generally, Nitisols are the dominant reference soil groups in coffee-growing areas of south west Ethiopia and study areas in particular [22].

Reliable climatic data of the area is not available due to a lack of weather stations for many years. Based on the information from the nearby stations at Gore, Tepi, and *Mizan Teferi*, the mean annual rainfall is estimated to be well over 2200 mm. The mean maximum temperature is estimated to be between 25°C and 34°C, and the mean minimum is estimated to be between 10°C and 15°C.

The rainfall distribution is uni-modal, with the highest rainfall between June and September. Rain falls throughout the year, with monthly minimum and maximum of about 70 and 220 mm. It also has a relatively long growing season of well over 250 days per year [24] and receives high amounts of rainfall, with an average between 1800 to 2200 mm per annum [25]

Ethiopia constitutes two of the 34 global biodiversity hotspots, namely the Eastern Afromontane and Horn of Africa biodiversity hotspots [24]. Southwest Ethiopia is the area where both Afromontane and Eastern biodiversity hotspots are located and composed of a number of diverse fauna and flora species. The Southwestern Ethiopian Afromontane rain- forests are the center of origin and diversity for wild Coffea Arabica. The areas have also constituted four biosphere areas (*Kaffa*, *Yayu*, *Sheka*, and *Majang*).

The existing high-value bio-diversity within the areas is the main factor for the demands of conservation activities. The livelihood of a majority of the communities within the area is profoundly dependent on the forest resources which have been upheld by systems of indigenous conservation practices called "*kobo* system". However, due to pressure from the outside as well as numerous other causes, it is increasingly challenging for these environmentally friendly indigenous cultures and practices to endure and be passed down through the generations. This necessitates maintain environmentally sound methods of forest management and conservation practices called the "*kobo*" system.

**Fig 2:**
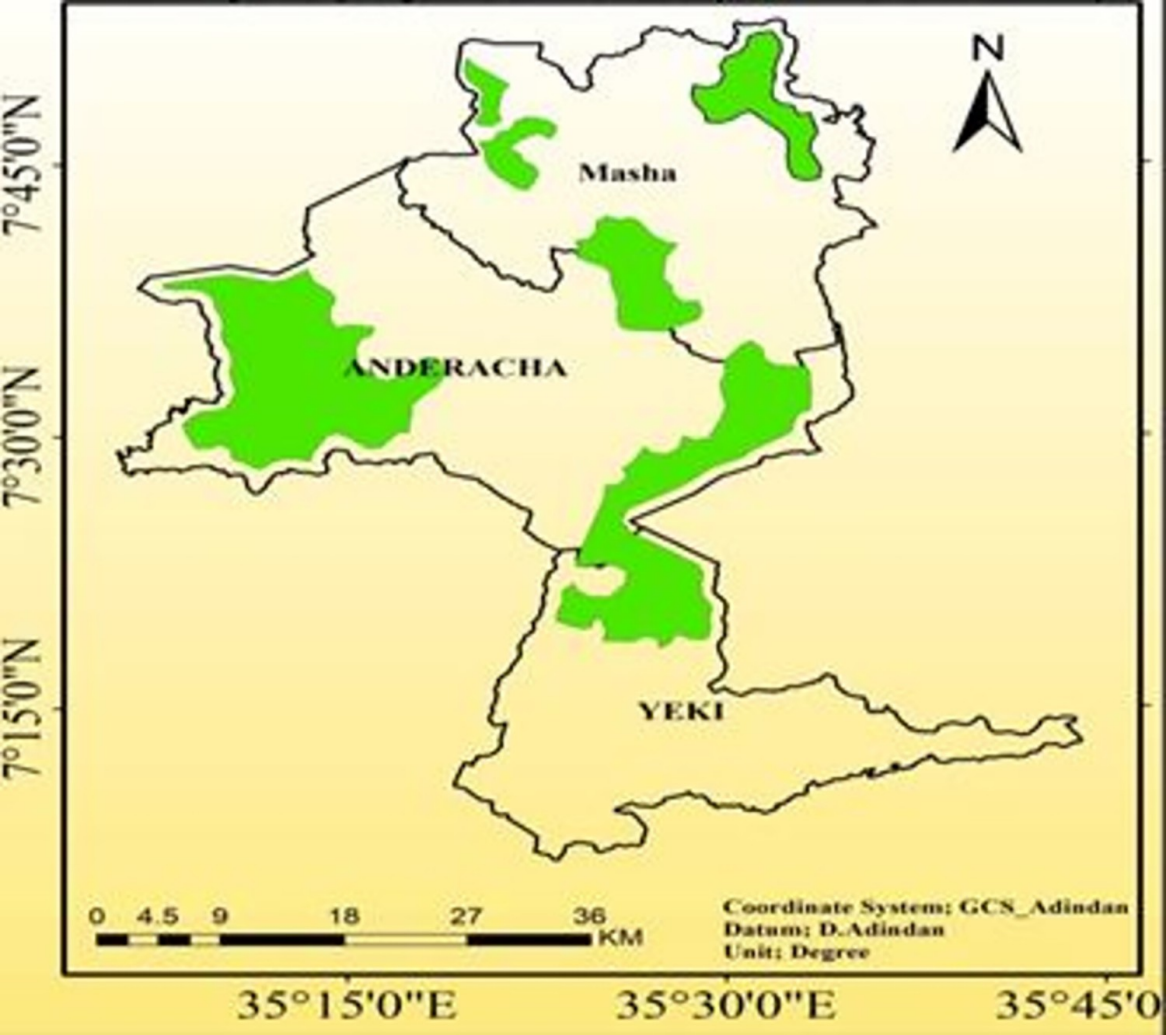
Map of biosphere reserve of *Sheka* zone (core area). Source; *Sheka* zone Forest and Environment protection department.

The figure below, "Fig 2," reveals that six locations were designated by UNESCO as core sites. These locations contain a total of 52,606.8 ha of dense forest, from which a core zone of approximately 12395.1 ha of dense forest was established from *Masha* woreda. The *Shato* core area is one of the three core sites in this woreda, with total area coverage of 5023ha.

**Fig 3:**
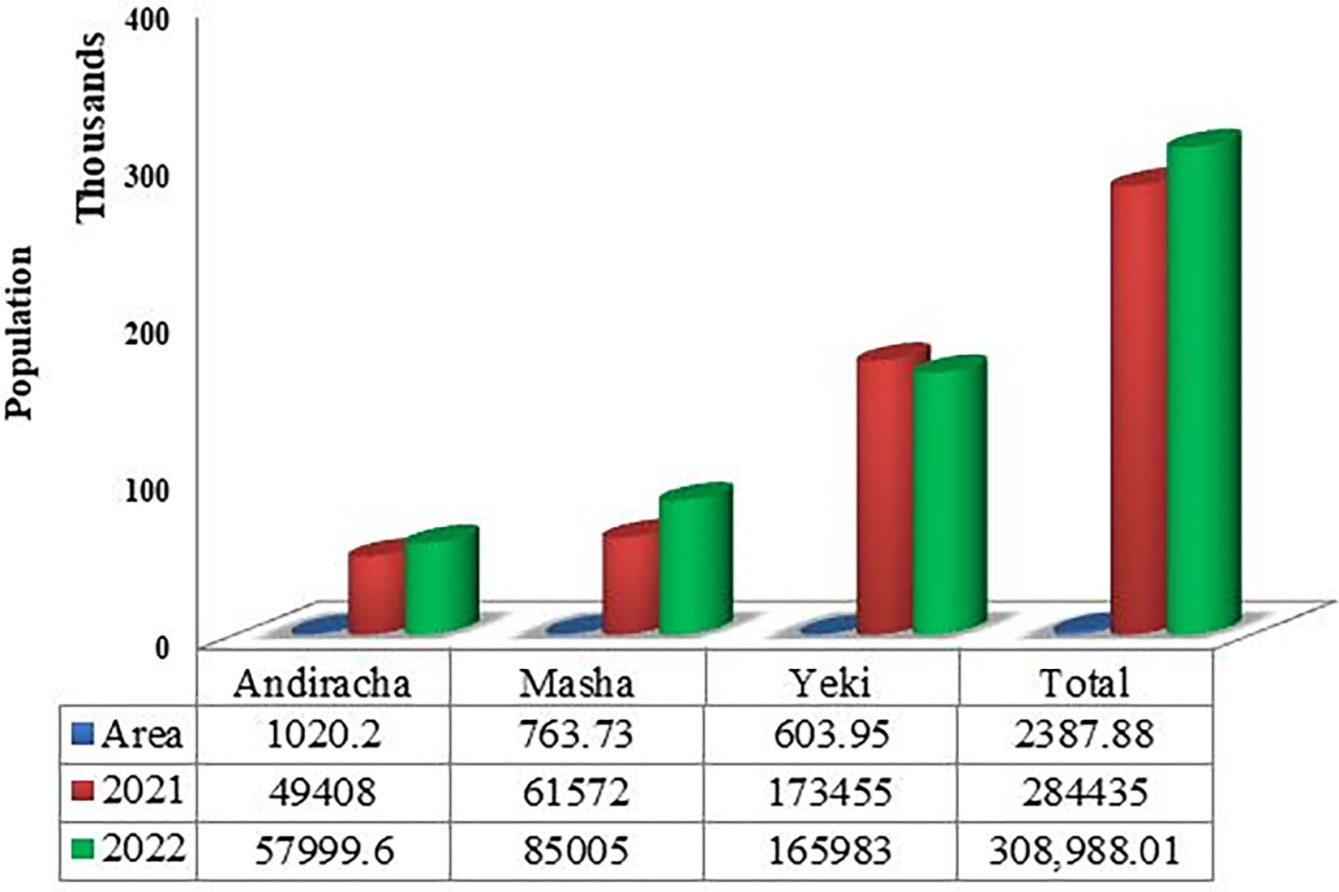
Graph of the total population of *Sheka* Zone (2021–2022). Source; *Sheka zone finance and economic development department (2022)*.

The Sheka zone has a population of 308,988, while the Masha woreda has a population of 85,005 (or 27.5% of the zonal population), according to projected demographic estimates from the *Sheka* zone finance and economic development department (2022). Masha woreda is the second-most densely inhabited woreda in the zone after *Yeki* woreda, which has a population density of 274.8/sqkm. Masha woreda has a total area of 763.73 sq km (Fig 3). Total population of yeki woreda have decreased from 173,455 in 2021 to 165,983 in 2022, because of existing of insecurity before a year, inhabitants were displace to surrounding *Woreda*s. This finding was purposively selected five kebeles of *Masha Woreda* such as; *Shay Limat (chewaqa)*, *Keja*, *Welo*, *Beto* and *Yepho*. Due to their proximity of two massive plantation projects, which contribute to a high rate of forest degradation, and the fact that these kebeles share a common boundary with the UNESCO-listed *Shato* core area, the most degraded site in the zone. A total of 11, 404 people, or 13.4% of the total population of the study area, reside in the five kebeles. The populations *of Keja*, *Welo*, *Beto*, *Yepho*, and *Shaylimat* are 3,392, 4,981, 1,008, and 1,113, 845, respectively.

The majority of Masha’s workforce is young and is engaged in agricultural activities. Cereals such as honey, enset, corn, grains, teff, beans, peas, and various spices are the main subsistence crops. Among them, the arable land area represents about 23.9%, grazing land represents 2.8%, forest land 40.5%, cultivated land 5.5%, non-arable land 5.9%, and settlement lands 21.4% [22]. Surveys conducted in this area show that honey, *enset*, livestock, annual crops, sugarcane, vines, *chat* and *gesho*, cardamom, wild coffee, palm, banana and *timiz* are the main means of survival, in descending order of importance [26]. The area is also famous for its important meat and dairy products from goats, sheep, dairy cows, and other domestic animals. In addition to agricultural activities, there are also transactions among rural residents in the small market in kebele, where people buy and sell coffee, honey and other products.

### 2.3. Data collections

**Fig 4:**
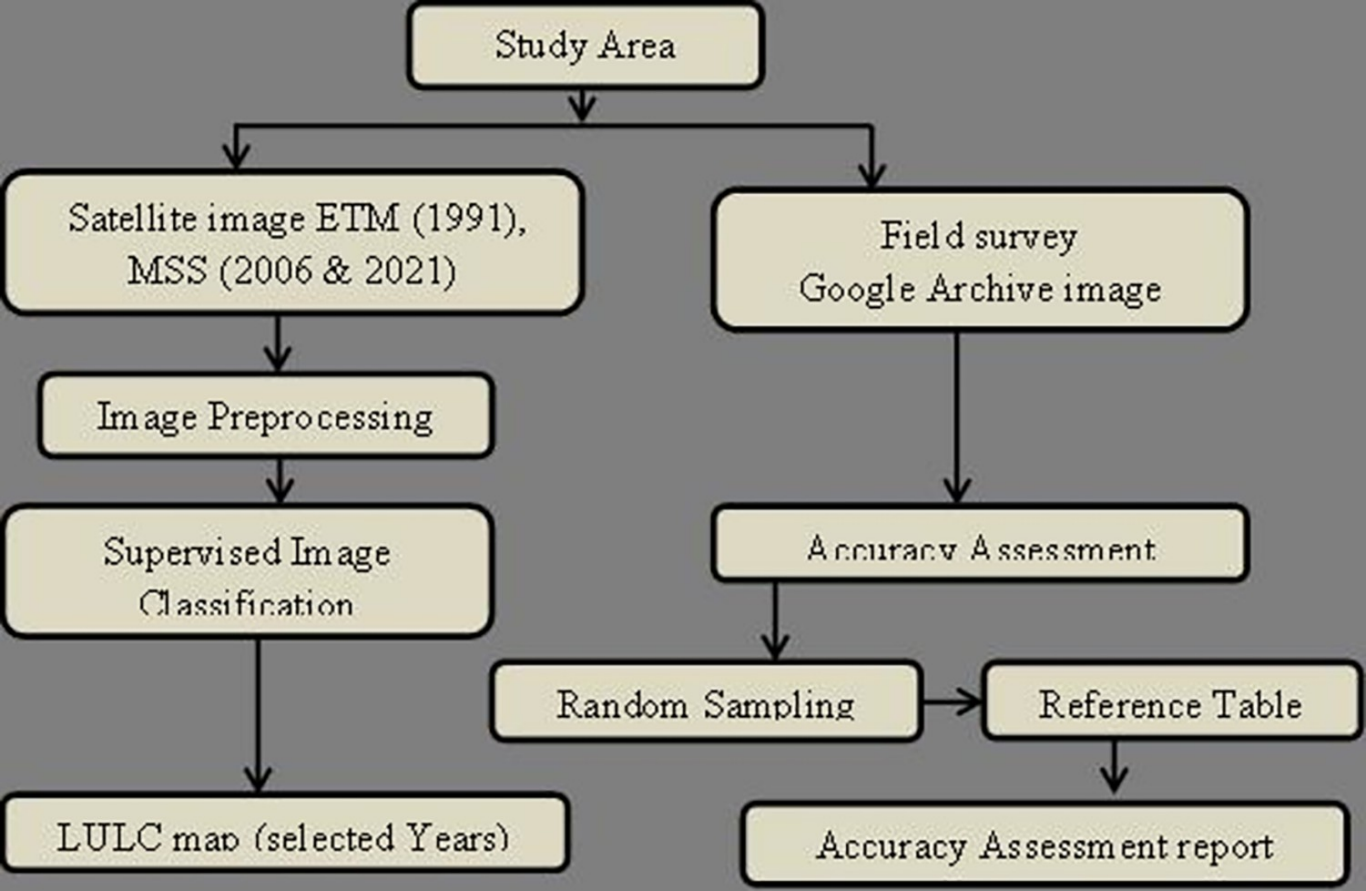
Schematic flow chart for land use/land cover dynamics and accuracy assessments. Source; developed by the author.

This study covers two sections: Land use/Land cover classification and accuracy assessment. The land use/cover classifications of the study area and accuracy assessment were carried out as per the methodology presented in “Fig 4”. *Sheka* zone forest, environment protection and climate change department gave permission for field work on study site.

#### 2.3.1 Image pre-processing

The classification process and analysis of the different LULC classes were done using three Landsat images downloaded from United States Geological (USGS) Earth Explorer with path and rows of 170 and 55 respectively. The researcher used Landsat images ETM of 1991, 2006 & 2021(MSS) from January up to March (relatively fewer cloud coverage months). The potential applications of Landsat ETM images are useful for image interpretation for a much wider range of applications than Landsat MSS images. This is due to the fact that the Enhanced thematic mapper (ETM) has more spectral bands than Multispectral scanner (MSS) does, as well as better spatial resolution. The interpretation accuracy of ETM data is improved due to the fewer mixed pixels that are present due to the reduced Instantaneous field of view (IFOV).

Since all Landsat images downloaded from the archive were already ortho-rectified, as a first step, due to having a different spatial resolution than the other image data, the 1991 ETM image is resampled to 30m to be conformed to the same resolution as 2006 and 2021 MSS imagery.

Intensive pre-processing such as spectral adjustment, mosaic, and layer stacking were carried out in ERDAS imagine software. The satellite image of each band was stacked in ERDAS Hexagon within interpreter main icon utilities with layer stacked function. Then, from the stacked satellite image the study area image was extracted by clipping the study area using ArcGIS 10.8 software. Besides, all images were checked and geo-referenced again as Adindan UTM Zone 36N.

After applying the above necessary steps, the researcher employed supervised image classification with the Non-Parametric Rule. The main advantage of supervised learning is that it allows expert involvement to collect data or produces a data output from previous experience and will have an exact idea about the classes in the training data. A practical minimum of 10N training pixels per spectral class is recommended, with as many as 100N per class if possible [27].

Vector layer (boundary, road & river) generated from google earth pro. While boundaries of investment (plantation and biosphere reserve) were obtained from *Sheka* zone forest, environment protection and climate change department. Finally, projected population density data is obtained from *Sheka* zone finance & economy department.

Both, Landsat images and Ancillary data were used to detect the trend of LULC dynamics in the study area. Ground control points were collected from the field for accuracy assessment. Software that was employed for mapping, accuracy assessments and Pairwise comparisons are; Arc GIS (V10.8), ERDAS (V2015) and IDRISI Salva.

#### 2.3.2. Sampling and sample size determination

Remote sensing involves two types of sampling; Remote sensing sampling and ground sampling [28]. To fulfill this objective the researcher used both remote sensing and ground sampling.

And, also employed, both temporal and spatial remote sensing sampling for the following reasons; temporally, to select satellite images of the initial and final year for LULC classification (1991–2021) and to identify cloud-free months of the year and acquire the images for (January up to march). Spatially, the resolution of the image determines the details of LULC classification. So, in this study, 30x30m resolution Landsat images were used.

Since remote sensing data is typically not obtained by the researcher, ground sampling needs direct involvement from the researcher or their team. Remotely sensed images nearly always need to be used in conjunction with reference data, sometimes known as "ground truth," to infer conditions regarding the earth’s surface [28]. In accordance with the results of the supervised classification, the researcher collected representative samples for each class of land use and land cover. As a result, there is a representation of ground control points in each class.

#### 2.3.3. Digital image processing and image classification

Digital image processing and classification is one of the fundamental steps in LULCC modeling. This method was primarily used for satellite image calibrations, which include histogram equalization, focal analysis, and noise and haze reduction. Using the ERDAS imagine 2015 software, specifically the median filters method, noise and haze were removed from the image.

The "correct" or "most appropriate" classifier is ultimately a subjective decision that reflects both the research objectives and characteristics of the data [29]. For the success of this study, the researchers were applied maximum likelihood classification techniques. Because, It is also one of the most popular methods of classification in remote sensing, in which a pixel with the maximum likelihood is classified into the corresponding class [30]. It is also one of the most common supervised classification techniques used with remote sensing image data, and was the first rigorous algorithm to be employed widely. Based on the field sampling data, the land cover types in the study area were divided into four categories; Forest, wetland, rural settlement, and plantation (coffee & tea).

#### 2.3.4. Description of land use/land covers types

As there are some differences between Land use/Land cover classes in the historical land-cover maps of 1991, 2006, and 2021 and land-cover classes, which can be discriminated from the satellite image, recoding was needed to create a common classification for change detection purposes.

This section describes the land classes, which are only used for land-cover mapping from satellite images

**Forest**; Land covered with trees reaching 5 m in height, 0.5 ha in area, and a canopy cover of >10% [31].

**Plantation** (tea & coffee); is defined as the crops cultivated in a vast area continuously grown and managed by an individual or a company. It was established in cleared forestland or modified forests [32].

**Rural settlements** are those areas composed of intensive use with much of the land by rural villages, towns, and roads. Or areas that are characterized by an artificial cover that replaces the original (semi-) natural cover. Also, a number of close families live in proximity to each other with fields surrounding the collection of houses and farmlands.

**Wetland**; is an ecosystem that depends on constant or recurrent, shallow inundation or saturation at or near the surface of the substrate. Wetlands are lands transitional between terrestrial and aquatic systems [33]

## 3. Result and discussions

### 3.1. Result

#### 3.1.1. Land use/land covers conversion analysis

Landsat data were divided into LULC groups and categorized using visual interpretation and the spatial data were verified using visual interpretation of Google Earth Pro images and training regions (ground truths) derived from repeatedly reviewed field data. To track changes in LULC, maps for remote sensing were created. Where appropriate, Arc GIS v.10.8 software was utilized. The percentage assessment of the proper classification was calculated by comparing the spatial data from the field.

To measure changes in land use and land cover, 1991 was used as the starting point. Three LULC conversions; between 1991 and 2006, 2006 and 2021, and 1991 and 2021, were discovered. The results of the changes in land use and the land cover showed that the first (1991–2006), second (2006–2021), and third study periods had significant losses and gains in LULC. Due to the expansion of investment-induced settlements in the study region and the growth of plantations (tea and coffee), the study region experienced significant changes in land use and land cover between 2006 and 2021. The “Table 1” 16,694.87ha (85%) of the study area was covered by forest and followed by 2,422ha (12.3%) and 543ha (2.8%) of rural settlements and wetlands respectively. It can be understood that the area was predominately covered by natural forest.

**Table 1:** Shows land use land cover class of study area for the year 1991.

No,	LULC types	Area(ha)	Percent (%)
1	Forest	16, 694.87	84.9
2	Rural Settlement	2, 422.02	12.3
3	Wetland	543.09	2.8
4	Plantation	1.02	0.005
Total		19661	100

**Fig 5:**
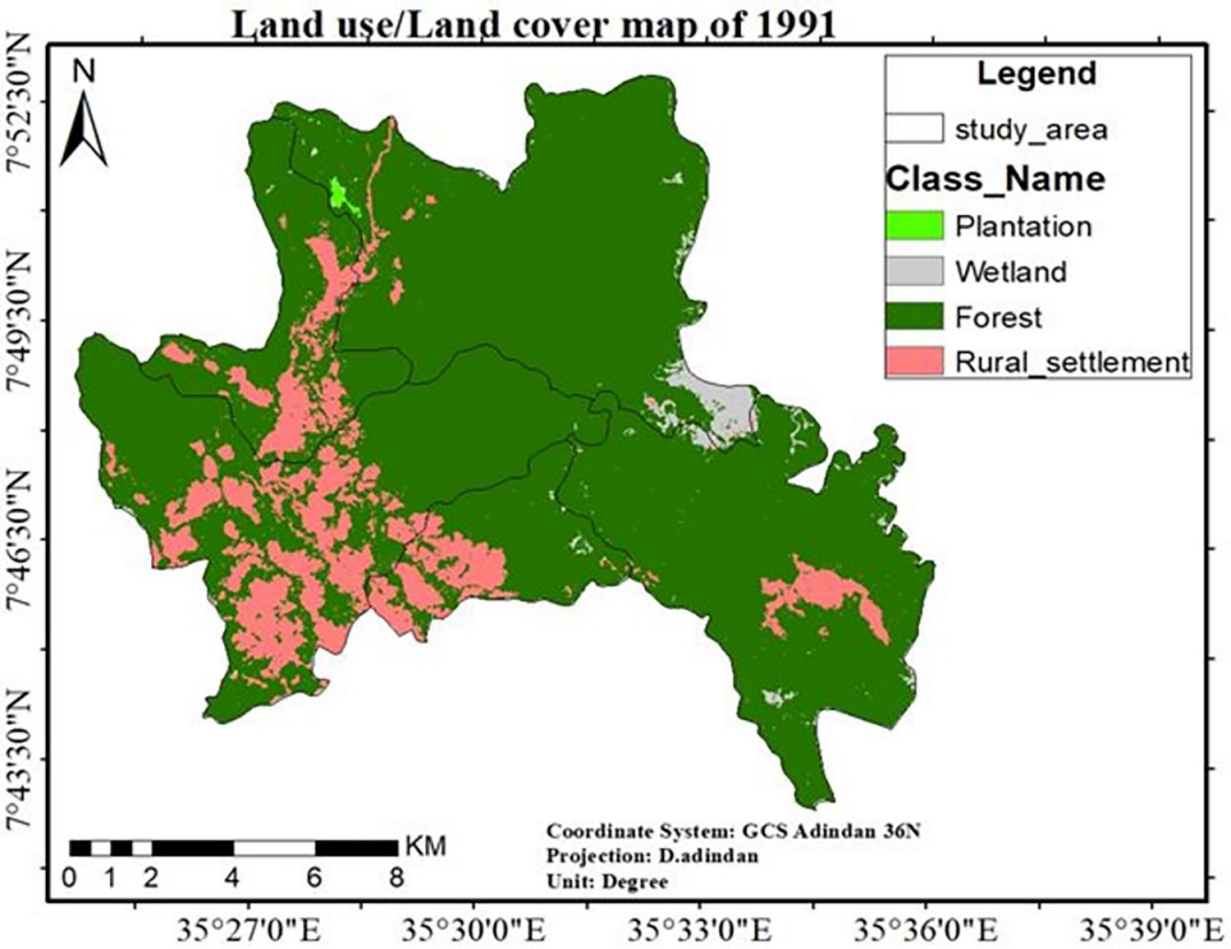
The LULC map of the study area for the year 1991. Source; Arc gis result of 1991 LULC classification.

The “Fig 5” shows, from five kebeles of the study area, *Chewaqa* was fully covered by natural dense forest and wetland, while in *Wollo* kebele forest coverage was relatively degraded because of expansions of rural settlement.

Although the forest coverage was still high, it declined by 838.9ha and the wetland also decrease by 104.4ha, while investment particularly tea plantations emerged in the study area in 1999 and took 692ha of natural forest in the first phase and because of investment induced population increment, rural settlement expanded by 251ha.

According to "Table 2” there were 438.71 hectares (2.2%) of wetlands and 15,857.1 ha (80.7%) of forest cover in the research area in 2006. Each type of land cover was reduced by 837.7ha and 104.38ha, respectively. While, rural settlements and plantations were expanded to 251.14 ha and 691 ha, respectively.

**Table 2:** Areal and percentage of land use land cover class of study area for the year 2006.

No,	LULC types	Area(ha)	Percent (%)
1	Forest	15, 857.11	80.7
2	Rural Settlement	2, 673.16	13.6
3	Wetland	438.71	2.2
4	Plantation	692.02	3.5
		**19, 661.00**	100

**Fig 6:**
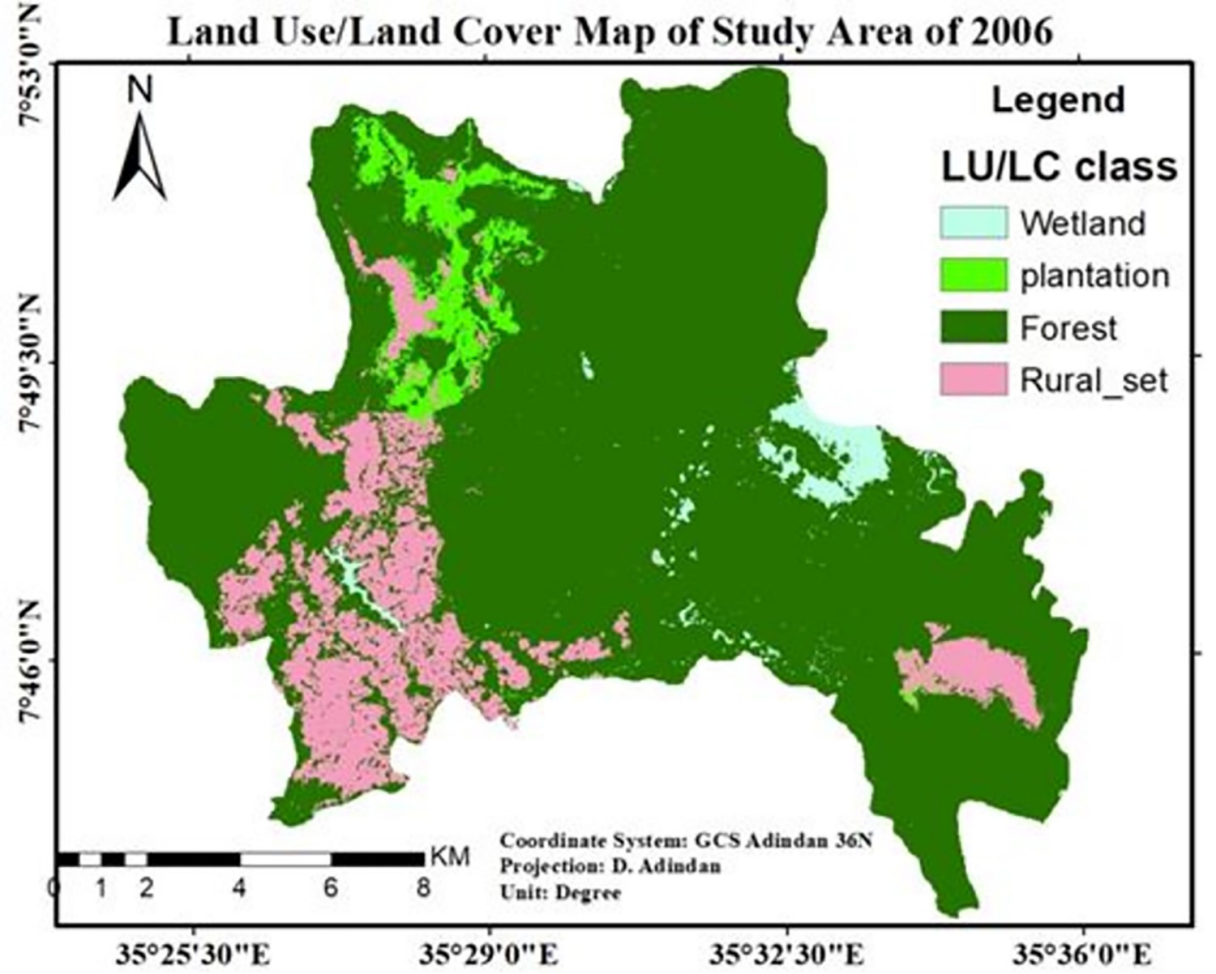
The LULC map of the study area for the year 2006. Source; Arc gis result of 2006 LULC classification.

The map shown in “Fig 6” that plantations emerged at the expense of natural forests at *Keja-Chewaqa* kebele and which pave the way for the emergence as well as expansion of settlements in and around these kebeles.

Because of tea and coffee plantation, and investment-induced expansion of rural settlement respectively, 2,234.3ha and 1,289.59ha of natural forest was cleared in the last 15 years (2006–2021). This shows that 320.35ha of natural forest was degraded each year. The following table “Table 3” will clearly explain the extent of forest cover change in the study area.

**Table 3:** Land use land cover class of study area for the year 2021.

No,	LULC types	Area(ha)	Percentage (%/)
1	Forest	13,480.30	68.6
2	Rural Settlement	3, 711.61	18.9
3	Wetland	234.88	1.2
4	Plantation	2,234.30	11.4
		**19, 661.09**	100

**Fig 7:**
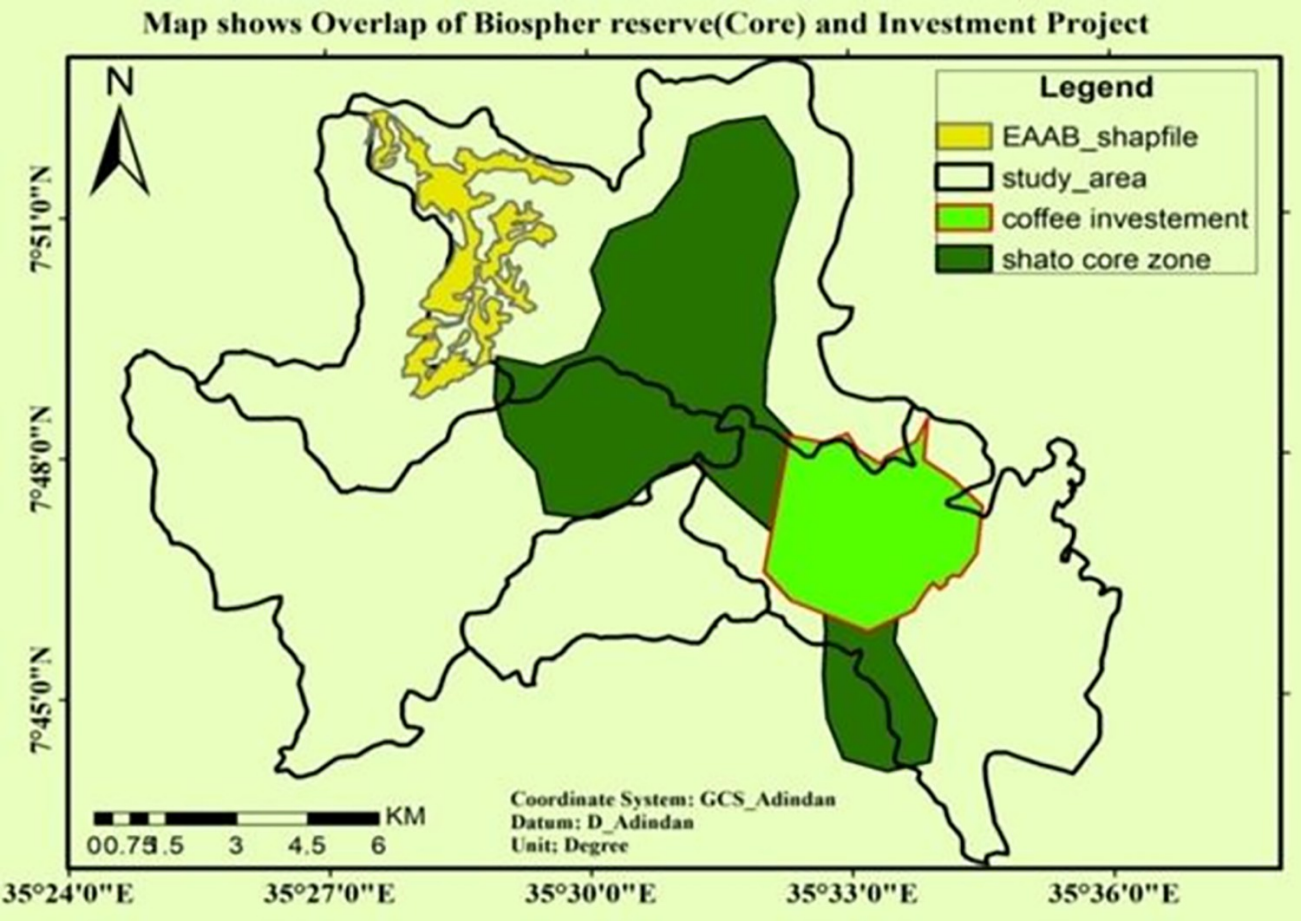
An overlap of biosphere reserve and investment. Source; Sheka zone Environment & forest protection department.

The following map, shown in "Fig 7," illustrates that overlap of the investment site and the biosphere reserve, which was designated as a core zone and is known as *Shato*. Due to investment opportunities, there was also observable human encroachment toward the core zone, which opened the door for the establishment of additional settlements close to the core area.

**Fig 8:**
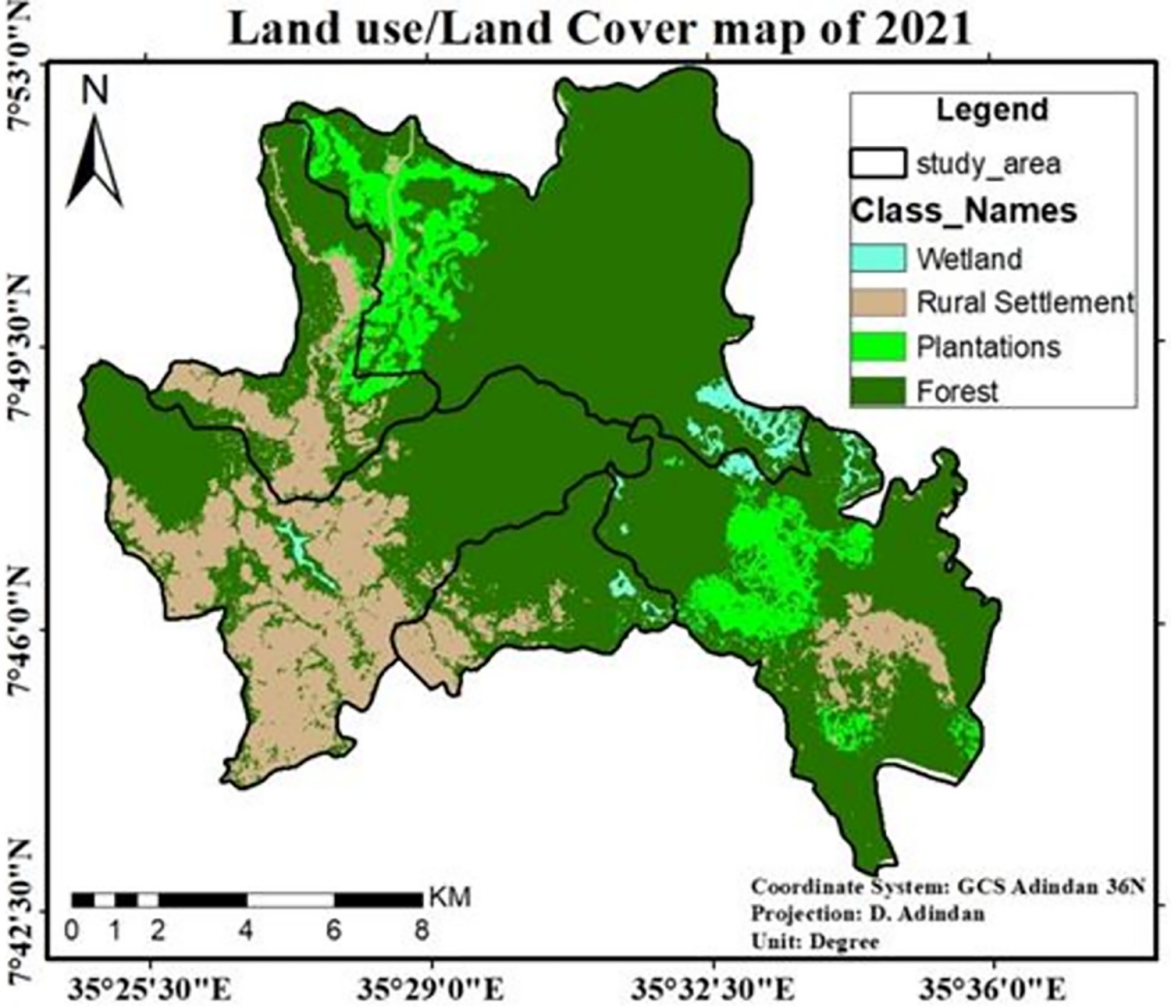
The LULC map of the study area for the year 2021. Source; Arc GIS result of 2021 LULC classification.

Depending on the data shown in “Table 4” the wetland was already vanishing, because above 308.29ha (56.7) of existing wetland and 1.6% of the total area was changed into other land use types in the last 30 years. And, 3,215.6ha (19.3%) of natural forest is also changed into rural settlements and plantations as compared with the initial period. The most shocking tragedy that happened to natural forests in the study area was almost all deforestation occurred in and around the core area of a biosphere reserve. The following map “Fig 8” will show clearly the severity of forest cover change in *Shato* core area.

**Table 4:** Land use/land cover change of study area for the years 1991, 2006 and 2021.

	LULC type								
Year	Forest		Rural settlement		Wetland		Plantation		
	Area(ha)	%	Area(ha)	%	Area(ha)	%	Area(ha)	%	Total
1991	16, 695.9	84.9	2, 422.02	12.3	543.09	2.8	1.02	0.005	19, 661
2006	15, 857.1	80.7	2, 673.16	13.6	438.7	2.2	**692.04**	3.5	19, 661
2021	13. 480.3	68.6	3, 711.61	18.9	234.8	1.2	**2, 234.3**	11.4	19, 661

#### 3.1.2. Accuracy assessment

It is an unavoidable step of LULC mapping, as the increased complexity of classification increases the chance of error [34]. However, a major constraint of this process is the availability and access to different map sources to cross-check the validity.

In this study, the accuracy assessment is performed by using ground truth points collected from the field and integrated with the high-resolution Google Earth imagery from Google Earth Pro. Three Google Earth imageries of March 1991, 2006, and 2021 are taken to validate the LULC maps of the same corresponding years. Besides, ground control points for March 2021 were collected from the field. By using the random sampling method, a total of 155 points were selected from different LULC classes in ArcGIS v10.8.

Error matrix is a common tool that is used to compare the pixel or polygon of the classified image with the ground truth data [35]. The matrices reflect the overall accuracy and the Kappa coefficient value for each year.

Various measures of accuracy assessment such as producer accuracy, user accuracy (35), overall accuracy, and Kappa coefficient were done. Overall accuracy [34, 36] was calculated as “**Eq 1”**, while Kappa coefficient [34] was calculated using “**Eq 2”**

$$OA=(\frac{x}{y})*100$$ Eq 1

Where OA is overall accuracy, X is number of correct values in the diagonals of the matrix, and Y is total number of values taken as a reference point.

$$K=\frac{N\sum_{i=1}^{r}xii-\sum_{i=1}^{r}\left(xi+*x+i\right)}{N^{2}\sum_{i=1}^{r}\left(xi+*x+i\right)}$$ Eq 2

In general, Kappa = (total accuracy–random accuracy) / (1- random accuracy). It can take values ranging from 0 to 1

According to the data acquired using the random sample technique shown in Table 5, the accuracy assessment of this finding was 74%, 81%, and 81.2%, respectively, for the images from 1991, 2006, and 2021. The accuracy of the user for all classifications varied from 60% to 88%, whereas the accuracy of the manufacturer varied from 60% to 91%. The wide range of accuracy suggests that wetlands and other classifications of land cover are seriously confused. Additionally, the producer accuracy measure (Sensitivity) represents the precision of the category’s forecast. Reliability of the classification to the user is shown in the User’s accuracy. The most accurate indicator of the classification’s real field utility is user accuracy. Forest was found to be more dependable, with a user accuracy of 88% in the year 2021.

**Table 5:** Accuracy assessment of the 1991, 2006, and 2021 classified images.

	Year					
	1991		2006		2021	
Class name	User accuracy	Producer Accuracy	User accuracy	Producer Accuracy	User accuracy	Producer Accuracy
Plantation	-	-	1	0.8	0.83	0.8
Rural Sett	0.8	0.7	0.83	0.83	0.83	0.8
Wetland	0.7	0.6	0.83	0.71	0.6	0.6
Forest	0.7	0.8	0.69	0.8	0.88	0.9
Overall accuracy	**74%**		**81%**		**81.20%**	
Kappa coef.	**0.74**		**0.81**		**0.812**	

An accuracy value greater than 70% is considered to be acceptable and the Kappa value ranging from 0.40 to 0.85 represents good correspondence [34]. Because, of the quality of +ETM 1991 image and cloud coverage, the kappa coefficient result of 1991 accuracy assessments 0.74 (which falls in good), while MSS 2006 & 2021 accuracy assessments results 0.81 and 0.812 respectively shows the best estimator of Land use/Land Cover classification in the study area.

#### 3.1.3. Transition matrix of land use/land cover change

**Fig 9:**
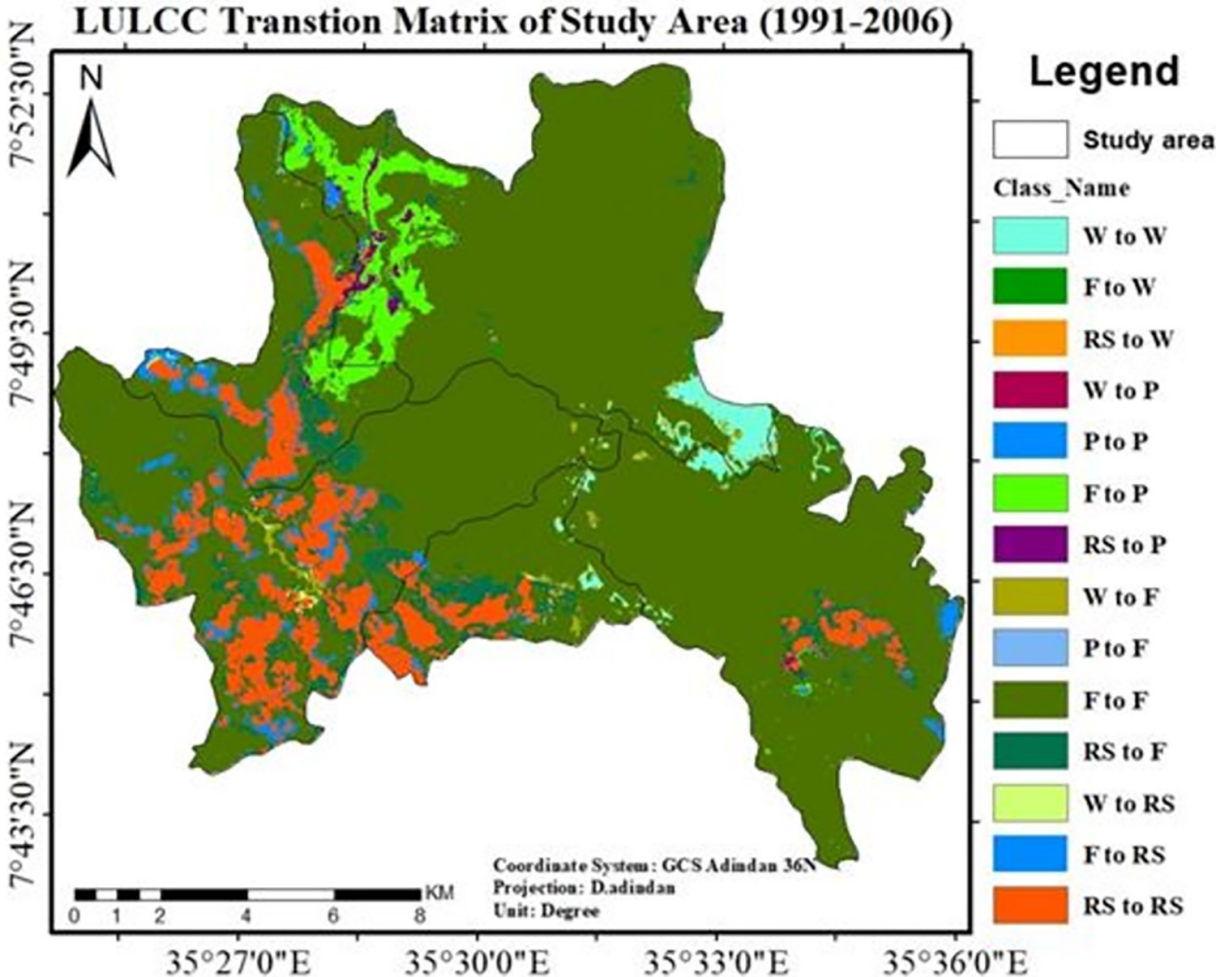
Transitions LULC change matrix of study area (1991–2006). **legend;** Where W; wetland, F; forest, Rs; rural settlement and P; plantation. **Source**; from the result of IDRISI salva Land change modeler.

In the year 1991, 84.9% of the research area was covered by natural forests and it was used as the baseline map. However, between 1991 and 2006, 74% of the forest persisted, whereas the rest was converted to plantations (6.2%) and rural settlements (4.5%). Additionally, 81.6% of the total loss of the forest occurred in the study area (1991–2006). According to the Land use/Land cover change matrix, plantations accounted for roughly 58% of the forest loss during this period, while rural settlements accounted for 41.8%. From "Table 6" and "Fig 9," it was deduced that between 1991 and 2006, the majority of the forest was degraded as a result of the creation of private plantations (East African Investment Group). This resulted in both the emergence of new settlements and the expansions of already-existing ones around the tea industry.

**Table 6:** Transition matrix of land use/land cover change (1991–2006).

		**2006**										
		Wetland		Plantation		Forest		R.Settle		Total	Loss	%
**1991**		Area(ha	%	Area(ha)	%	Area(ha)	%	Area(ha	%			
	Wetland	**428.5**	2.2	61.4	0.3	37.6	0.2	37.2	0.2	567.4	136.2	0.7
	Plantation	4.8	0.02	**1.0**	0.0	2.9	0.01	3.2	0.02	12.0	11.0	0.1
	Forest	9.5	0.05	1217.4	6.2	**14,533.4**	73.9	880.7	4.5	16721	2107.6	10.7
	R.settle	7.7	0.04	92.6	0.5	79.0	0.4	**2180.2**	11.1	2360.5	179.3	0.9
	Total	450.5	2.3	1372.4	7.0	14653.0	74.5	3101.3	15.8	**19661**	2434.0	12.4
	Gains	22.0	0.1	1371.4	7.0	119.6	0.6	921	4.7	2434		

Similar to the years 1991 to 2006, forests account for 65.9% of all losses (2006–2021). The result in "Table 7" reveals that plantation and rural settlement constituted the majority of the conversion during this time.

**Table 7:** Transition matrix of land use/land cover change (2006–2021).

		2021										
		Wetland		Plantation		Forest		Ru. Settlement		Total	Loss	%
		Area(ha)	%	Area(ha)	%	Area(ha)	%	Area(ha)	%			
**2006**	Wetland	**17.1**	0.09	234.2	1.2	175.7	0.9	11.6	0.06	440.7	421.5	2.1
	Plantation	0.2	0.001	**918.1**	4.6	154.7	0.8	58.2	0.3	1136.6	213.1	1.1
	Forest	118.0	0.6	1025.5	5.1	**13,338.8**	66.8	849.1	4.3	15404	1992.6	77.2
	Ru.Settle	6.0	0.03	77.9	0.4	310.9	1.6	**2283**	11.4	2,679.7	394.7	13.4
	Total	141.3	0.71	2255.6	11.3	13,980.1	70	3,201.9	16	19661	**3021.9**	
	Gains	124.2	0.6	1337.5	6.7	641.3	3.2	918.9	4.6	**3021.9**		

**Fig 10:**
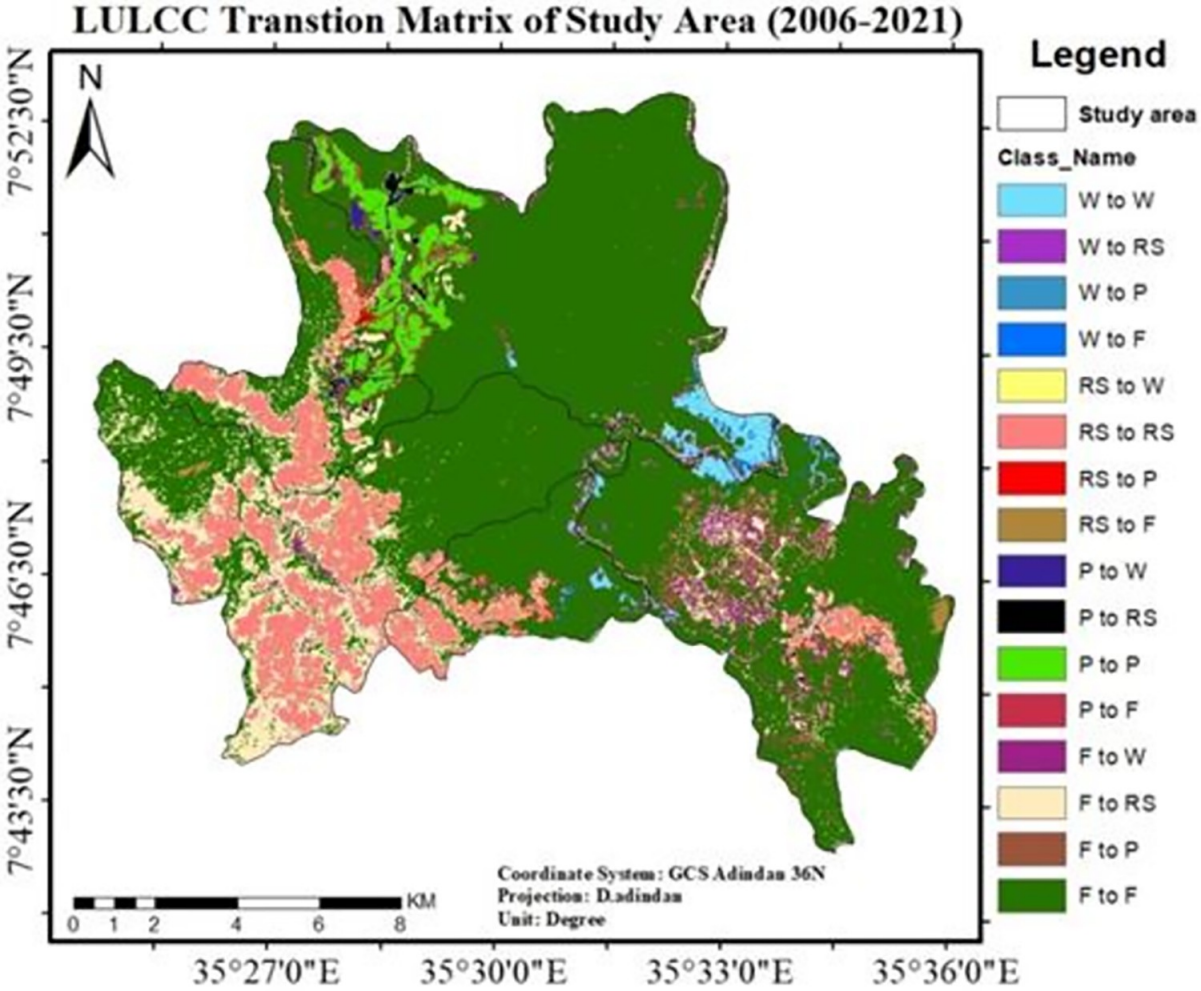
LU/LCC transition matrix of the study area (2006–2021).

Moreover, during this time a new coffee plantation known as Haile Coffee Investment Plc. emerged, resulting in the loss of 1482 hectares of natural forest. Devastating damage was also done to the natural forest in the core area (*Shato*) "Fig 10". This kind of catastrophe is a likely the result of replacing natural forests with tea and coffee intensive agriculture.

**Fig 11:**
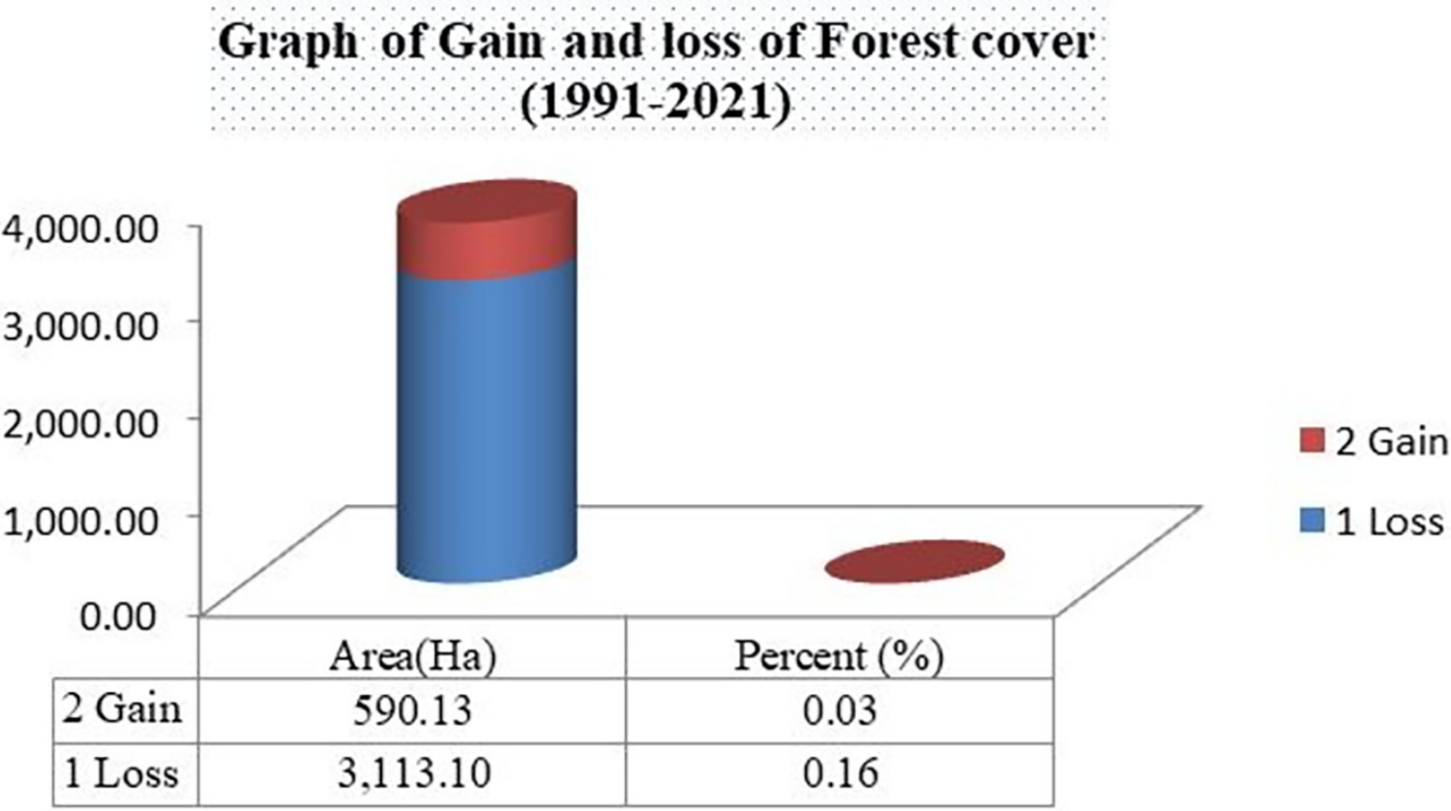
Gain and Loss of Forest cover of the study area (1991–2021). Source; from the result of IDRISI Salva Land change modeler.

**Fig 12:**
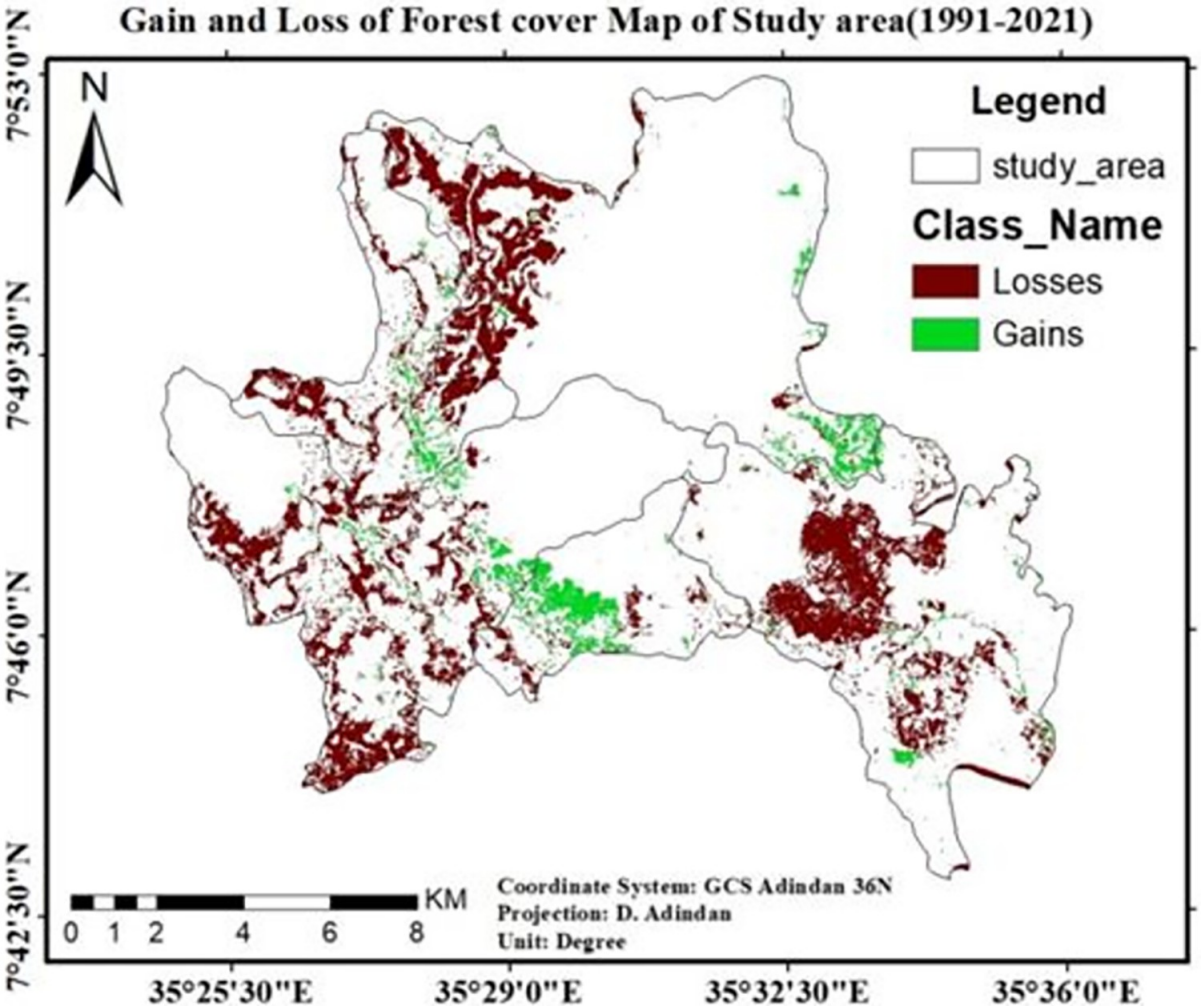
Loss & gain of forest cover of the study area (1991–2021). Source; from the result of IDRISI Salva Land change modeler.

The bar graph in “Fig 11” and map in “Fig 12” implicates that from 1991–2021 average forest cover of the study area lost by 3,113.1ha and gains only 590.3ha. So, the last 30 years forest cover of study area decreases by 2,522.97ha (84.1ha/yr).

**Fig 13:**
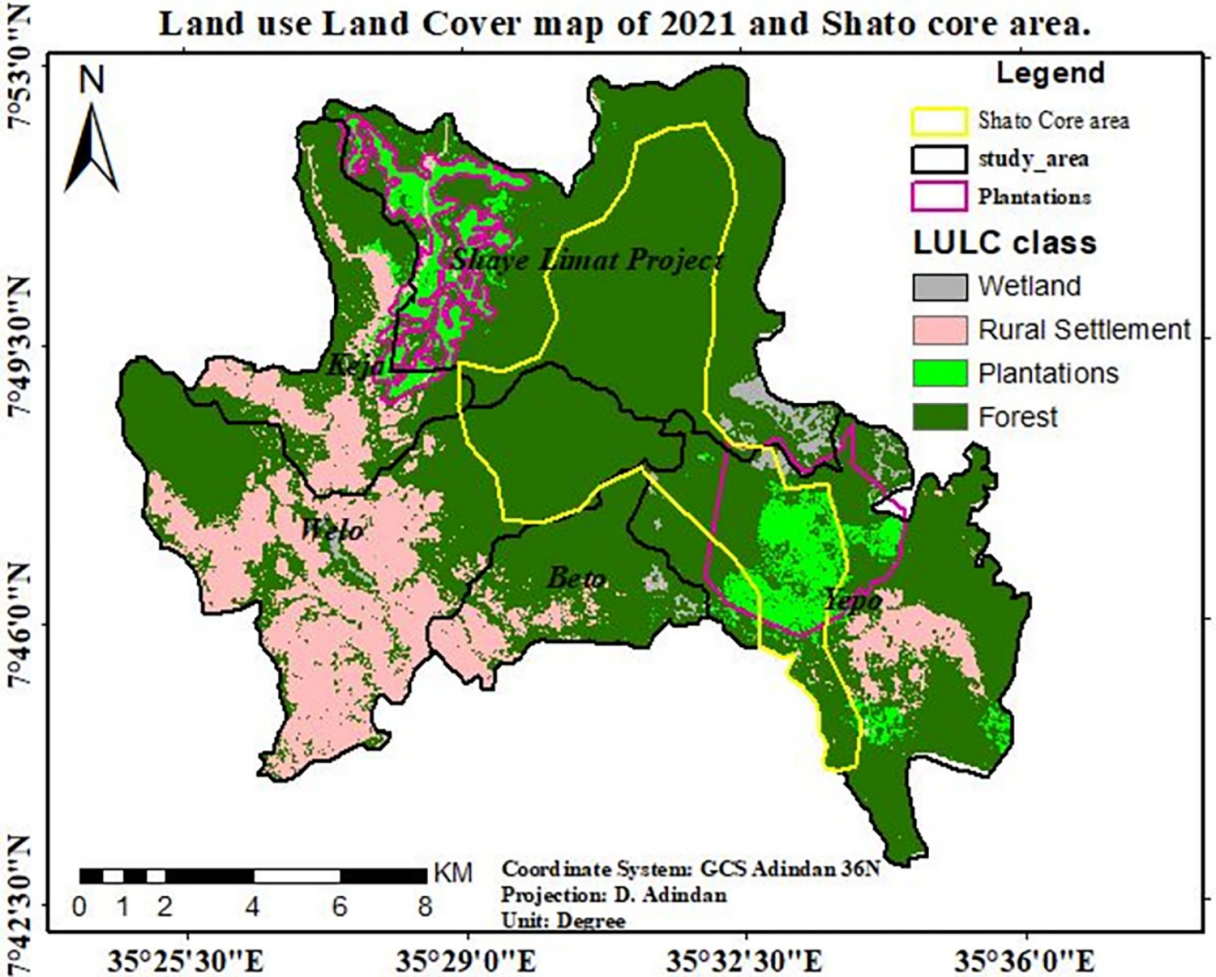
Land use/Land cover map of 2021 and *Shato* core area. Source; Arc gis and *Sheka* zone forest, environment protection and climate change department.

The majority of natural forest loss in the last 30 years is intensified in areas around plantations. Three kebeles such as; *Shay Limat*, *Keja*, and *Yepho* were seriously affected by plantation aggravated forest degradations, which paves the way for the establishment, as well as expansions of rural settlements around the area, occupies by private owned plantations (tea & coffee). Forest degradation undoubtedly has a detrimental effect on the state of biodiversity [37]. One of the shocking facts was that the coffee plantation in the core area’s center received roughly 1500 hectares of natural forests ("Figs 12 and 13"). The status of biodiversity in *Shato* core is therefore found to be seriously endangered by the expansion of both coffee plantations and rural settlements.

**Fig 14:**
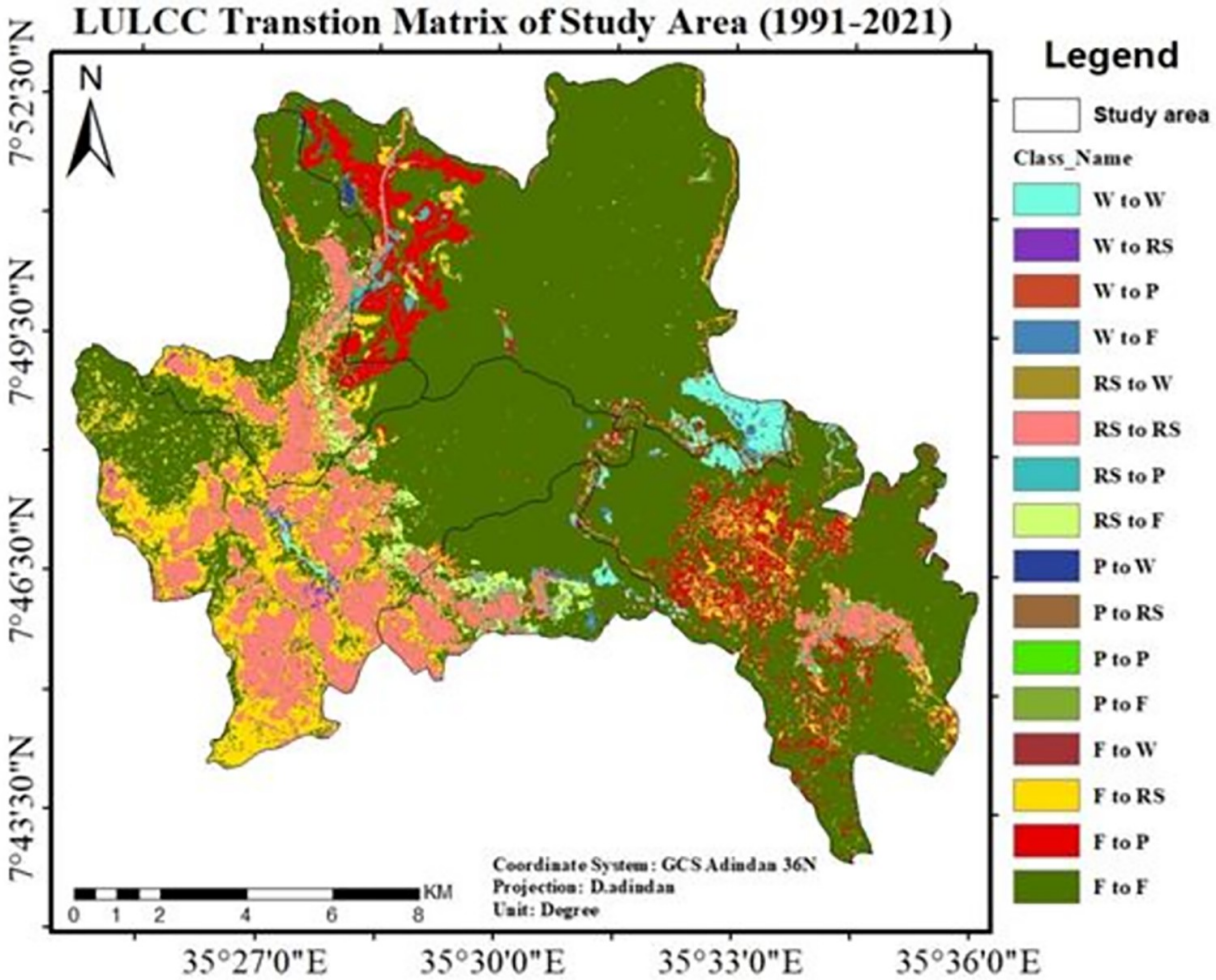
Land use/Land cover change transition matrix of the study area (1991–2021). **legend**; Where W; wetland, F; forest, Rs; rural settlement and P; plantation. Source; from the result of IDRISI Salva Land change modeler.

The transition matrix of LULC dynamics in the study area is depicted on a map in "Fig 14" (1991–2021). It shows that the area marked in red represents the conversion of natural forest and wetland into plantations, while the area highlighted in yellow and pink represents the conversion of forest to rural settlements.

### 3.2. Discussion

A major element of LULC concerns is the interaction of people with their surroundings [38]. Such an interaction covers a broad range of resources, stakeholders, and institutions (at various levels). The vulnerability of areas and individuals to climatic, economic, or socio-political changes is also influenced by changes in land use and cover [39].

The pressure from anthropogenic activities and climate change is changing and/or affecting ecological services more and more. This diminishes the ability of individuals in developing countries, where direct dependence on the natural environment is still quite strong, to adapt. It also restricts the delivery of ecosystem products and threatens livelihoods [40].

Due to the combined effects of several factors, the forest cover in the southwestern biosphere reserves has been decreasing at an alarming rate. Deforestation’s underlying causes are associated with a variety of anthropogenic and natural sectors [31].

The natural environment has been under intense strain from humanity. For instance, the growth of human settlements has expanded as a result of the increasing human population[41, 42].].

Increasing adverse changes in land use and land cover have major effects on the food security and livelihoods of the local population as a result of environmental degradation, climate change, expanding human populations, resource extraction, and other developments [43, 44]. A deeper conception of the intricate relationships between environmental changes and shifting rural people’ livelihood strategies is required in light of these massive changes.

This study applied remote sensing techniques to classify satellite imagery of the *Shato* core area and its surrounding, *Sheka* biosphere reserve 1991 to 2021. Our objective was to identify the locations, types, and trends of the major LULCs during the 30 years. Although the land cover was directly observed in the field, observations of land use and its changes were generally not straightforward. Where the issue is less about the natural and more about anthropogenic drivers and sustainability of woody specious biodiversity in the face of human pressures, records have also been utilized to good effect.

This study showed that there were rapid LULC changes in three decades (1991–2021) in study sites “Fig 14”, with plantation and rural settlements replacing forests. Land use/land cover change analysis of the study area was run, and maps were generated for the last three decades. Depending on the areal extent, quality, and resolutions of Landsat image and dominant land use types practiced in the study area, a total of four LULC types were identified in the study area in all study periods from 1991–2021.

This study has quantified the dynamics of LU/LCC and its drivers in *the Shato* core area of the *Sheka* biosphere reserve south western Ethiopia. The result showed that about 20% of natural forest in the study area was converted to other LU/LC types while plantation and rural settlement increased by 2, 234.3ha (10.2%) and 1289.6ha (6.6%).

*Shato* core area covers 5023.3ha (25.5%) of the study area, and currently, only 3,541ha (18%) are left. The rapid decline in forest resources occurred between 2006 and 2021 “Table 7” and “Fig 10”.

As shown in “Figs 5–7”, forestland accounted for the largest proportion of wetland, plantation, and rural settlement. Forestland and wetland were decreased and mainly transformed into the plantation and rural settlements in all study periods “Table 6”. This is possibly due to agricultural expansion (particularly large-scale private investment) leading to human population increment in the study site. The expansion of large-scale investment in the *Sheka* zone was an agent for the degradation of natural forests [11].

The classification result of 2021 “Table 3” shows that only 66.8% of forest land was left. The result of this finding was consistent with other studies carried out in different parts of the country, for instance, [45] in the *Assosa* area of southwest Ethiopia stated that only 74% of the forest cover persisted and the rest was converted into farmland between 1957 and 1995. The overall accuracies were attained by Landsat +ETM (0.74), and MSS (0.81), for the years 1991, 2006, and 2021 “z5”. The values of overall accuracy and kappa values above 81% indicate that the classification performance is satisfactory [46].

The observed trends of increasing tea and coffee plantations, rural settlement, and decreasing forest land in the area could be explained by: First, investment-induced work opportunities lead to an increment of the rural population in the study area and leads to the search for more land for settlement. Second, the population growth forced the farmers to till and expand their lands to a greater extent than before to cope up with the conditions and sustain their life. Third, infrastructure expansion at the expense of forest land and wetland has contributed to the reduction of those land use/ land cover types in the area.

Earlier studies at the national level and in *Sheka* Forest revealed that many people were giving up unproductive agricultural land found nearby. Rural settlement has also shifted and settled in and around the core area, and the pressures of an increasing population have an impact on the degradation of natural resources [14, 47]. Fragmentation and diminishing of the natural vegetation, and hence the native species habitat, are the direct effects of land cover change [37, 48]. The researcher was observed that, in addition to the efforts made by various researchers on the causes of LULCC that have been done in the *Sheka* zone [10, 14, 49], the main driving forces behind in the *Sheka* biosphere reserves (*Shato* core area in particular) were commercial plantation (coffee and tea by investors), agriculture expansions, settlement, fuelwood extraction, illegal logging for timber, and farm tools. This study revealed that human factors, such as the expansion of settlements and plantations, have primarily caused the loss of potential natural forests and biodiversity. The major proximate drivers of LU/LCC were an expansion of subsistence and commercial agriculture and unsustainable exploitations of forest products. The increase in population from natural increase and immigration, lack of proper execution of legal and institutional arrangements related to agricultural development, and natural resources management are the major underlying causes of LU/LCC in the study area.

## 4. Conclusion

The main data sources needed for planning and decision-making are land use/land cover change analyses. To comprehend the historical and contemporary circumstances of the studied area, an integrated method was adopted in this study.

The study’s key findings include the following: employing Landsat images to create thematic LULC maps for change comparison and dynamics. There were found to be four LULC classes. Based on the study’s findings, an analysis of LULC classification change for the study’s period revealed dynamism and discovered that there has been a rapid increase in rural settlement and agricultural land, particularly large-scale plantations, while there has been a downward trend in forest land and wetland.

The forest cover and its biodiversity status are in danger as a result of the widespread occurrence of poor land resource management, the removal of vegetative cover, population development and the following expansion of large-scale farming, as well as rising resource demand. The principal conclusion of this research is that, among other things, the change in cover in the study area may have an effect on natural resources and livelihoods of communities who depend on forest and perceive it as being essential to providing food and income.
