# Correction: Trends of Land Use Land Cover Dynamics of *Sheka* Biosphere Reserve, A Case of *Shato* Core Area, Southwest Ethiopia

**DOI:** 10.1371/journal.pone.0296112

**Published:** 2023-12-14

**Authors:** Workaferahu Ameneshewa, Yechale Kebede, Dikaso Unbushe, Abiyot Legesse

The affiliation for the first author is incorrect. Workaferahu Ameneshewa is not affiliated with #1 but with #2: Department of Geography and Environmental Studies, College of Social Science, Arba Minch University, Ethiopia.
